# Implementation of Health Policies in the COVID-19 Pandemic Phases of Myanmar and How the Population Approach Influenced Their Success Rate

**DOI:** 10.7759/cureus.50944

**Published:** 2023-12-22

**Authors:** Khine Zin Aung, Han Thiri Zin, Sa Tin Myo Hlaing, Putri Damayanti, Tamanna Tabassum

**Affiliations:** 1 Biostatistics, University of Kentucky College of Medicine, Lexington, USA; 2 Biomedical Sciences, Hood College, Frederick, USA; 3 Public Health, Faculty of Medicine, University of Miyazaki, Miyazaki, JPN

**Keywords:** developing countries, public health policy, infection spread, public health care, covid 19

## Abstract

The impact of the COVID-19 pandemic is continuing in developing countries, and post-pandemic individuals are still suffering mentally and physically. Many researchers have tried to find the causes and risks that can impact the spread of disease. Among the causes and risks identified, socioeconomic factors and health policies played an important role in determining the transmission of the disease. However, the significance of these factors for the spread of infection is different depending on the country. In this editorial, we discuss the implementation of health policies in Myanmar and their effect on infection transmission.

## Editorial

Myanmar, a developing country in Southeast Asia, has suffered from many impacts of COVID-19 because of its vulnerability in healthcare and socioeconomic conditions [1]. The details of COVID-19 in Myanmar are available for the period August 20, 2020, to January 31, 2021. According to a previous ecological study in Myanmar, population density and medical-related variables were found to be associated with COVID-19 transmission [1]. More studies are needed to find out the effective countermeasures against infectious diseases in the future.

To prevent the spread of infectious diseases, the primary countermeasures are 1) blockage of infection sources such as lockdown, contact tracing, and disinfection; 2) checking the infection routes and taking necessary precautions; and 3) countermeasures, especially for susceptible people such as elders and immunodeficient people [2]. However, the approaches to countermeasures are different in different countries [3, 4]. In developing countries, the population approach is still a gold standard because it can easily be applied to populations with limited resources. Therefore, health policy settings by the government to reach the population level became important in the infection prevention of COVID-19.

**Figure 1:**
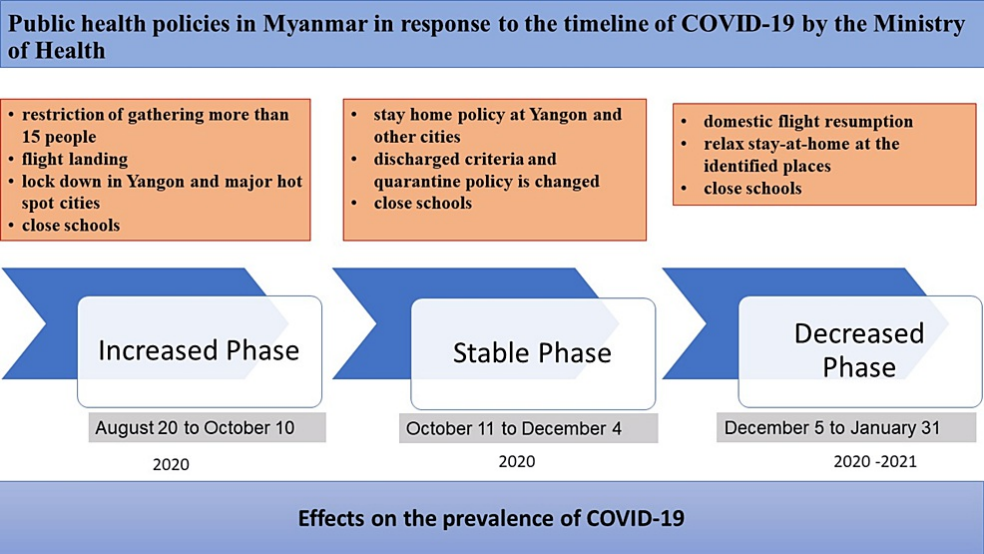
Summary of public health policies in Myanmar during 3 phases of COVID-19 infection by Ministry of Health, Myanmar. Myanmar's public health policies during the three stages of COVID-19 infection. Policy information was obtained from the Ministry of Health, Myanmar's publicly accessible official website [5].

The timeline of COVID-19 infection in Myanmar was divided into an increasing phase (August 20, 2020 to October 10, 2020), a stable phase (October 11, 2020 to December 4, 2020), and a decreasing phase (December 5, 2020 to January 31, 2021) [1]. Yangon, which is the commercial and most populated city, had the highest cases of COVID-19. The Ministry of Health (MOH) in Myanmar and government officials also announced public health policies in each phase of COVID-19 transmission (Figure 1) [5].

In this editorial, we discuss the effectiveness of health policies by population approach in Myanmar in each phase. In the increasing phase, the policies included banning gatherings of more than 15 people, restrictions on international flight landings, lockdowns in major economic cities, and school closings. In the stable phase, the MOH announced the stay-at-home order in hot spots for COVID-19 transmission, quarantine policies were changed according to WHO guidelines, and schools were still closed then. In the decreased phase of COVID-19, the policies were relieved and only carried out in affected areas [5].

The overview of these policies gave insights into the effectiveness of primary countermeasures with a population approach toward population density by observing the period (from stable to decreased phase) and case reduction in those phases in Myanmar. For example, the range of cases of COVID-19 per 10^4^ population in the stable phase is 0.76-67.9, and in decreased phase is 1.0-22.2 [1]. The previous ecological study in Myanmar concerning COVID-19 spread factors indicated population density was the main factor that was found to be associated with the transmission of infection. Other factors like income, employment, aging, and transportation were not significantly correlated and the policies we discussed also did not focus on those factors. However, the policies have some limitations to some hard-to-reach areas, details concerning individuals such as virology status, host defense, and comorbidities. Several studies also reported that population density is an effective factor against COVID-19 and policies for social restrictions in developing and developed countries [3,4].

In conclusion, even in developing countries like Myanmar, health policies focusing on population density are still effective against infection outbreaks. We hope this discussion can give insights into future infection control in the primary phase of infection in developing countries with limited health care and resources.
